# Oncological-Therapy Related Oral Mucositis as an Interdisciplinary Problem—Literature Review

**DOI:** 10.3390/ijerph17072464

**Published:** 2020-04-03

**Authors:** Aida Kusiak, Barbara Alicja Jereczek-Fossa, Dominika Cichońska, Daniela Alterio

**Affiliations:** 1 Department of Periodontology and Oral Mucosa Diseases, Medical University of Gdansk, 80-210 Gdansk, Poland; dominika.cichonska@gumed.edu.pl; 2 Department of Oncology and Hemato-Oncology, University of Milan, 20141 Milan, Italy; barbara.jereczek@ieo.it; 3 Division of Radiotherapy, IEO European Institute of Oncology, IRCCS, 20141 Milan, Italy; 4 Division of Radiation Oncology, European Institute of Oncology IRCCS, 20141 Milan, Italy; daniela.alterio@ieo.it

**Keywords:** oral mucositis, oral cavity, oral mucosa, treatment

## Abstract

Oral mucositis is a toxic side effect of non-surgical cancer treatments: chemotherapy and radiotherapy, which strongly impair quality of life and can not only cause strong pain, but also lead to problems with basic physiological needs as eating and swallowing. Development of oral mucositis is associated with type, dosage, and schedule of radiation or chemotherapy and other factors related to patients. Management of oral mucositis is a valid problem, requiring topical application of anesthetics, coating agents, cryotherapy, low level laser therapy, pharmacological methods as usage of keratinocyte growth factors, supplementation of vitamins, and a proper diet. Another approach to oral mucositis measurement includesphotobiomodulation, which brings analgesic and anti-inflammatory effects.Although oral mucositis is a general health issues, the role of proper dental care is essential. It should include elimination of all potential sources of mucosal injury and microorganisms inhabiting theoral cavity through oral hygiene education, professional management ofdental plaque, and treatment of the caries and periodontium, which are necessary to reduce the risk of inflammation in the oral cavity. This paper describes the possibilities of monitoring oral mucositis, taking into account the latest therapeutic achievements.

## 1. Introduction

Chemotherapy and radiotherapy are widely used methods of non-surgical cancer treatments for head and neck tumors which prolong life or even completely treat the disease. However, they cause numerous toxic side effects, including oral mucosa disorders [1,2], which strongly impairs quality of life [3]. The most widely observed disorderis oral mucositis, which concerns 75% of high risk patients treated with radiotherapy or high-dose chemotherapy [1,4]. Otherwise, according to Sonis, almost all patients who receive chemotherapy and cumulative radiation doses of >30 Gy are likely to develop oral mucositis [5], whereas Elting at al. claim that clinical evidence of radiation-induced oral mucositis is 80–91% [6]. Severe oral mucositis might be the cause of unplanned hospitalization or even lead to treatment changes including breaks in radiation [7] or reduction of chemotherapy dosage [8], resulting in negative effects on treatment outcome [9,10]. Management of oral mucositis requires a holistic approach, including cooperation with dental practitioners, to provide patients with a proper care.

Aim of the present study is to present an interdisciplinary overview on oral mucositis in patients treated for head and neck tumors.Therefore, a descriptive review of the literature onpathogenesis, risk factors, diagnosis, and treatment strategies has been performed.

## 2. Pathogenesis and Risk Factors of Oral Mucositis

The process of oral mucositis development is very complex and includes five phases: initiation, primary damage response, signal amplification, ulceration, and healing [11]. The initiation of oral mucositis requires DNA damage which causes lack of proliferation ability in epithelium basal cells. However, even more important is generation of reactive oxygen species (ROS) [12,13,14]. In primary damage response the activation of transcription factors is observed, which results in upregulation of pro-inflammatory cytokines, cytokines modulators, adhesion molecules, stress responders, and matrix metalloproteinases. This leads to thinning of the epithelium and oral mucosa destruction caused by tissue injury and cell death [15,16]. Signal amplification is the ability of some of the molecules present in previous phases to potentiate and strengthen tissue injury [11]. However, development of ulceration is the most significant mucositis stage due to the fact that it is highly symptomatic for the patient. Ulcers are colonized by both gram-positive and gram-negative bacteria present in the oral cavity [15]. Products of bacterial metabolism may exaggerate damage of the oral mucosa and stimulate additional release of pro-inflammatory cytokines. Healing of ulcerative lesions in oral mucositis is spontaneous and requires a series of biological processes appearing on the submucosa layer [15,16].

Risk factors of oral mucositis might be divided to treatment- and patient-related categories. However, even the tumor itself can be highly significant. Treatment-related factors are associated with the type, dosage, and schedule of the radiation or chemotherapy, drug selection, and potential usage of adjuvant agents [15]. Patient-related factors are older age, body mass index, oral environment, and genetic predispositions [15]. Female gender also plays a significant role, as it has been proven that female patients present higher risk of oral mucositis for methotrexate [17] and 5-fluorouracil (5-FU) treatments [18]. Oral mucositis is a disease which may also concern children;however patients under the age of 10 present less severe symptoms and shorter duration chemotherapy-related oral mucositis [19].

Genetic factors also play an essential role in development of oral mucositis. Firstly, genes pose an impact on the action of enzymes which metabolize chemotherapy drugs, for instance a defect of dihydropryrimidine dehydrogenase would increase the potential toxicity risk of 5-FU [15]. However, known enzymatic deficiencies regarding oral mucositis predisposition are rare.

## 3. Clinical Features of Oral Mucositis

Clinical manifestation of oral mucositis takes various forms and might develop from mucosal erythema to small lesions and ulceration. The most commonly used scale to measure symptoms of oral mucositis for both clinical and research purposes is the World Health Organization WHO scale [20]. (Table 1):

Another scale, divided into clinical examination and symptoms, was developed by the National Cancer Institute (NCI) [21] (Table 2).

The oral mucositis assessment scale by Olsen et al. differentiates oral mucositis by theamount of lesions, mucosal erythema, and presence of bleeding, and is presented in Table 3 [22].

Clinical features of oral mucositis can be also estimated by an oral assessment guide created by Eilers et al. which is presented in Table 4 [23].

At the beginning stages of oral mucositis, it clinically manifests only as a mucosal erythema, without any lesions or ulcerations, and patients complain only about a feeling of burning. Although such a form of oral mucositis appears at the beginning of chemotherapy or radiation, in some cases it may not transform to more severe stages [24]. However, the majority of patients develop more severe forms of oral mucositis, which manifest as deep and very painful ulcerative lesions of all oral mucosa, which preclude physiological functions as nutrition or swallowing. Ulcers occurring in oral mucositis have significantly different clinical manifestations than ulcers associated with aphthous stomatitis or any dental trauma. Borders of mucositis ulcers are difficult to define and we do not observe the peripheral ring of erythema, which is caused by lack of inflammatory components. The location of changes is uncharacteristic; erythema and ulcers associated with oral mucositis may appear in all areas of oral mucosa, but most frequently on buccal mucosa, the floor of the mouth, the soft palate, and borders of the tongue. In case of ulceration localized on the dorsal surface of the tongue, gingiva, or hard palate, other etiology might be suspected [24].

There are significant differences between oral mucositis induced by chemotherapy and head and neck radiation. Patients subjected to chemotherapy start to present symptoms one week after treatment and heal within two weeks. On the contrary, mucositis induced by radiation requires more time to evolve and heal. In that cases ulceration usually appear two weeks after the beginning of radiation and disappear around three to four weeks after the end of the radiation series [11,24]. Mucositis being a result of target therapies is believed to differ from both chemo- and radiotherapy induced mucositis and clinical manifestation of ulcers seems to be more like aphthosus stomatitis [25].

Mucositis is mostly associated with pain, which strongly decreases patients’ quality of life. This poses a negative effect on patients’ physical, emotional, and social comfort [26]. Increase of mucositis symptoms also presents negative correlation with patients’ ability to follow the treatment plan of the underlying disease and extends the time of required hospitalization [7].Patients suffering from oral mucositis often require hydration, parental nutrition, pain management, and hospitalization, whereas patients without symptoms of severe oral mucositis might be treated in an ambulatory setting [27]. Ulcerated mucosa is also prone to secondary infections, which may develop to systemic infections, which is another reason for longer hospitalization of patients suffering from oral mucositis [28]. These may lead to a costs increase of supportive care of patients undergoing chemo- and radiotherapy [27,28].

## 4. Material end Methods

A literature search for relevant papers indexed in the literature from 2011 to 2020 was conducted using PubMed and Scopus databases. In our paper we included only randomized studies concerning cryotherapy, photobiomodulation (PBM), and usage of palifermin or caphosol among patient with oral mucositis. Key words included: oral mucositis, management, prevention, cryotherapy, low level laser therapy, photobiomodulation, palifermin, caphosol, randomized trial. Low level laser therapy (LLLT) is an old term; however is a still-used form of treatment and a PubMed key word.

We have also screened the references of the systematic reviews and meta-analyses to identify additional, original studies that were not found in our prior search.

## 5. Management of Oral Mucositis

Proper management of oral mucositis is a key element to improve patients’ quality of life and reduce time of required hospitalization. Good basal oral health is significant in reduction of the risk and severity of oral mucositis [2,29]. All potential sources of mucosal injury should be eliminated before the beginning of chemo- and radiotherapy because they may exaggerate and extend development of oral mucositis [2,24]. Sharp surfaces of teeth must be restored, orthodontic appliances removed, and dental prosthesis used as rarely as possible [30]. Reduction in the amount of microorganisms inhabiting oral cavity is also an important issue. Oral mucositis is not an infectious disease, however secondary colonization of ulcerations poses a negative influence on mucosal healing processes [24]. The largest reservoir of microorganisms in the oral cavity is supra- and subgingival dental plaque which makes the periodontium a source for potential acute infections [29]. A professional management ofdental plaque is essential to reduce risk of inflammation in the oral cavity. Proper oral hygiene is also valid in this case, especially if salivary flow is reduced after radiotherapy. All patients should be provided with oral hygiene education [24].

Another aspect which may reduce the symptoms of oral mucositis is a proper diet. Tobacco, alcohol, and all acidic, spicy, and products containing processed sugar should be eliminated [3,8]. Strong pain associated with oral mucositis can made food intake difficult or even impossible. This is the reason why soft and liquid diets might be required. Patients who are expected to develop severe symptoms of oral mucositis sometimes undergo prophylactic placement of a gastrostomy tube [31].

Reduction of oral mucositis symptoms can be acquired by using special mouthwashes containing topical anesthetics and coating agents, antibiotics, angifungals, and steroids [32]. Topical anesthetics should ensure local pain control, however potential systemic side effects should be minimal. They may cause alteration of taste and should not be administrated as a prophylaxis [21]. Coating agents may also bring pain reduction by forming a protective coating on an ulcerated oral mucosa. However, this method is insufficient among patients with severe oral mucositis that require systemic analgesics, including opioids [33]. Corticosteroid can also be used due to their anti-inflammatory properties [34]. Management of oral mucositis may include usage of benzydamine mouthwash due to its anti-inflammatory properties, which inhibit production of TNFα and IL-1β [35]. According to Ariyawardanaet al.,benzydamine mouthwash is the only anti-inflammatory agent with evidence in prevention of oral mucositis [36]. Topical anesthetic agents are very common usedamong patients with oral mucositis and clinical experience suggests that they can be beneficial in some patients to provide temporary relief. However, topical morphine concentrations are not clearly defined and range from 0.08 for the gel to 0.2% to 2% for the rinse which can lead to a conclusion that this topic requires further evaluation [37].

Patients undergoing radiation often suffer from xerostomia and hyposalivation, which may increase the risk of local infection and impede mastication. Firstly, it is advised to sip water as often as possible and use artificial saliva or other supporting products. If these methods areinsufficient, cholinergic agents are required [31].

Treatment of oral mucositis may also include supplementation of vitamins A and E. Vitamin E, being an antioxidant, may cause a reduction of the severity of mucositis by decreasing the damage from oxygen radicals [2]. Vitamin A can restrain inflammation process and its decreased level among patients with severe oral mucositis was observed [34]. However, according to a systematic review by Yarom et al., there islimited evidence of vitamin efficiency in management of oral mucositis and in this case no guidelinesarepossible [38].

There are also natural methods of oral mucositis management including honey and herbal compounds. Topical application of honey has been observed to promote wound healing [39] and presents anti-microbial properties which may be useful in prevention of secondary infections [40]. According to a systematic review prepared by Yarom et al., there isclinical evidence that topical application combined with systemic administration of honey is beneficial in prevention of oral mucositis among patients treated with chemotherapy or head and neck radiotherapy [41].

In severe cases of oral mucositis induced by chemo- and radiotherapy, where proper dental care, diet, and usage of topical agents are insufficient, there are also other methods including cryotherapy, photobiomodulation (PBM), and usage of palifermin or caphosol.

Cryotherapy has also been proven to reduce symptoms of oral mucositis among patient undergoing chemotherapy, due to vasoconstriction and reduction of blood flow. It is believed that topical usage of ice chips fiveminutes before administration of the chemotherapy dosage reduces blood flow and decreases delivery of chemotherapeutics to oral mucosa [42].According to Askarifar et al., cryotherapy is more effective than saline mouthwash in reducing the severity of mucositis. This was a single, blinded, randomized clinical trial including 29 patients. Patients receiving cryotherapy developed less severe forms of oral mucositis [43]. Cryotherapy seems to be a promising method of oral mucositis management, however has not been well examined among patient undergoing chemotherapy and head and neck radiation yet. However, there are interesting randomized studies concerning the effectiveness of cryotherapy on oral mucositis induced by allogeneic hematopoietic stem cell transplantation. In a prospective randomized study conducted by Lu et al. on a group of 145 patients receiving allogeneic hematopoietic stem cell transplantation, the effectiveness of cryotherapy in oral mucositis management was proven. In this study patients were receiving cryotherapy from the beginning of a conditioning regimen infusion until the end (whole course), from the midpoint of a conditioning regimen infusion until the end (second half of the course), and as part of daily nursing practice. It was proven that cryotherapy could decrease the incidence and duration of severe oral mucositis and the results were comparable between groups receiving whole-course and half-course schedules. However, cryotherapy should not be used as a part of a daily routine care due to the potential damage of oral mucosa [44]. On the contrary, according to a randomized controlled trial conducted by Kamsvåg et al. on a group of 49 pediatric patients undergoing hematopoietic stem cell transplantations, no reduction in the incidence of severe oral mucositis after application of cryotherapy was observed [45].

Another approach to oral mucositis measurement includes photobiomodulation, previously named low level laser therapy, which has analgesic and anti-inflammatory effects and accelerates wound heling [46,47]. Prophylactic and therapeutic photobiomodulation can be used to reduce symptoms of oral mucositis. This is a topical application of a monochromatic, coherent light source, which presents a cytoprotective effect. In reduction of oral mucotitis symptoms, low level laser therapy should be used before and during oxidative stress connected with chemo- and radiotherapy [48]. According to meta-analysis conducted by Migliorati et al. [49], He et al. [50], and Bjordal et al. [51], there is a strong effectiveness of photobiomodulation in reduction of oral mucositis symptoms, including duration, severity, and pain. No serious side effects were reported [51]. In a randomized placebo controlled trial conducted by Gantam et al. on a group of 46 elderly patients with radiation induced oral mucositis, PBM decreased the severity of oral mucositis and also reduced usage of analgesics [52]. Another randomized placebo controlled trial, which was conducted by Oton-Leite et al. on a group of 30 patients with head and neck cancer, resulted in the conclusion that PBM was effective in reducing the severity of chemoradiotherapy-induced oral mucositis [53]. A randomized placebo controlled trial which was conducted by Antunes et al. on a group of 94 patients also proved that PBM is effective in preventing chemoradiotherapy-induced oral mucositis and improves quality of life [54]. Recent experimental studies conducted by Rezk-Allah et al. on a group of 80 patients proved that PBM pose a positive effect on chemotherapy induced oral mucositis and is well-tolerated by patients [55]. All patients with oral mucositis received PBM 6 days/week and the outcome parameters were levels of TNF-α and IL-6 measured before, during, and after administration of PBM. Usage of PBM results in improvement of oral mucositis; however, the mechanism of action does not seem to be related to the change of pro or anti-inflammatory cytokines and further research isrequired to fully understand the mechanisms of influence of laser therapy on oral mucosa [55]. According to a systematic review prepared by Zadik et al.,photobiomodulation is recommended for the prevention of oral mucositis [56]. However, according to a prospective study conducted by Guedes et al.,photobiomodulation with high doses of laser energy (1.0 J versus 0.25 J) causes only a small improvement in prevention and management of oral mucositis [57]. The effectiveness of photobiomodulation in oral mucositis management is difficult to estimate due to the fact that there are different therapy protocols used in conducted clinical trials.

There are also pharmacological methods of oral mucositis treatment. An agent approved to use in oral mucositis treatment is palifermin, keratinocyte growth factor-1. This is a protein produced by mesenchymal cells which stimulates cellular responses and is expressed almost exclusively by epithelial cells in a wide variety of tissues, including buccal mucosa [58]. Palifermin is clinically proven to reduce severity and duration of oral mucositis and improve quality of life [59,60,61,62]. In randomized and placebo-controlled studies conducted by Henke et al. on a group of 186 patients it was proven that palifermin not only decreased the severity and duration of oral mucositis, but also reduced the occurrence of this chemo-radiation side effect. In this study patients received weekly palifermin 120 μg/kg or placebo from 3 days before and continuing through out chemoradiotherapy [61]. Le at al. also concluded that palifermin reduced symptoms and severity of oral mucositis. In this randomized and placebo-controlled study, patients received palifermin (180 μg/kg) or the placebo once before starting chemoradiotherapy and then once weekly for 7 weeks [62]. Research by Henke at al. showed only mild side effects of palifermin and there were also no negative influences on treatment results. Furthermore, survival of patients receiving palifermin and the placebo were almost the same [61]. Moreover, according to Stiff et al., the long term safety outcomes among patients receiving palifermin in oral mucositis treatment and the placebo were comparable [63].

Another promising pharmacological method of oral mucositis treatment was caphosol, a mouthwash which is well-tolerated by patients and presents no side effects [64]. This is a calcium phosphate electrolyte solution which replaces normal ionic balance, modulates apoptosis, regulates mediators of pain and inflammation, and activates epithelial proliferation. According to Papas et al., statistically significant reduction in oral mucositis duration after usage of caphosol was observed [65]. However, other research did not confirm the effectiveness of caphosol. In a double-blinded placebo-controlled randomized trial conducted by Raphael et al. on a group of 29 patients, where 15 of them received caphosol, the therapeutic use of caphosol was not beneficial in the treatment of oral mucositis in comparison to the placebo [66]. A similar conclusion was presented by Rao et al. in a phase II multicenter trial conducted on 98 patients receiving head and neck radiation. Caphosol was taken by patients at least 4 times a day and up to 10 times per day beginning with day 1 of radiation and for a total duration of 8 weeks after completion of radiation. Caphosol did not significantly reduce oral mucositis classified as WHO grade 2 or higher [67]. Treiser et al., in a randomized double-blinded placebo-controlled clinical trial conducted on a group of 220 children and young adults, also did not observe a reduction of oral mucositis severity due to caphosol usage [68]. Caphosolwasalso examined by Svanberg et al. in comparison to cryotherapy. In this randomized study, the experimental group received cryotherapy combined with caphosol and control group received only cryotherapy. Cryotherapy was administrated from the start to the end of high dose chemotherapy and caphosolwas administered from day 0 to day 21. There were observed noadditional effects of caphosol usage [69].

A summary of randomized double-blind studiesincluding cryotherapy, photobiomodulation (PBM), and usage of palifermin or caphosol is presented in Table 5.

From the technological point of view, the recent developments of radiotherapy modalities have reduced the volume of the exposed healthy mucosa, decreasing the treatment toxicity [70].

## 6. Conclusions

Oral mucositis is a severe side effect of head and neck radiation and chemotherapy which highly impairs patients’ quality of life and can not only cause strong pain, but also leads to problems with basic physiological needs such as eating and swallowing. The pathogenesis of oral mucositis is well known; however more studies in the field of treatment are required. There are several methodsof oral mucositis management, but no specific guidelines have been created andclinical studies conducted in previous years present conflicting results.Although oral mucositis is a general health issue, the role of proper dental care is essential in management of this disease.

## Figures and Tables

**Table 1:** The World Health Organization (WHO)scale for oral mucositis.

**Grade 0**	No oral mucositis
**Grade 1**	Erythema and soreness
**Grade 2**	Ulcers, able to eat solids
**Grade 3**	Ulcers, requires liquid diet (due to mucositis)
**Grade 4**	Ulcers, alimentation not possible (due to mucositis)

**Table 2:** The National Cancer Institute (NCI) scale for oral mucositis.

Oral mucositis (clinical examination)	
**Grade 1**	Erythema of the mucosa
**Grade 2**	Patchy ulcerations or pseudomembranes
**Grade 3**	Confluent ulcerations or pseudomembranes; bleeding with minor trauma
**Grade 4**	Tissue necrosis; significant spontaneous bleeding; life-threatening consequences
**Grade 5**	Death
**Oral mucositis (functional/ symptomatic)**	
**Grade 1**	Minimal symptoms, normal diet
**Grade 2**	Symptomatic but can eat and swallow modified diet
**Grade 3**	Symptomatic and unable to adequately aliment orhydrate orally
**Grade 4**	Symptoms associated with life-threateningconsequences
**Grade 5**	Death

**Table 3:** Western Consortium for Cancer Nursing ResearchWCCNRstomatitis staging system.

Score	Lesions	Erythema	Bleeding
**0**	None	50% or more pink	None
**1**	1–4	50% or more slightly red	
**2**	>4	50% or more moderately red	With eating or mouth care
**3**	More than 50% denuded	50% or more very red	Spontaneous

**Table 4:** Oral assessment guide of oral mucositis.

Category	Tools for Assessment	Methods of Measurement	Numerical and Descriptive Ratings		
			1	2	3
**Voice**	Auditory	Converse with patient	Normal	Deeper or raspy	Difficulty talking or painful
**Swallow**	Observation	Ask patient to swallow. To test gag reflex, gently place blade on back of tongue and depress	Normal swallow	Some pain on swallow	Unable to swallow
**Lip**	Visual/palpation	Observe and feel tissue	Smooth and pink and moist	Dry or cracked	Ulcerated or bleeding
**Tongue**	Visual/palpation	Feel and observe appearance of tissue	Pink and moist and papillae present	Coated or loss of papillae with a shiny appearance with or without redness	Blistered or cracked
**Saliva**	Tongue blade	Insert blade into mouth, touching the center of the tongue and the floor of the mouth	Watery	Thick or ropy	Absent
**Mucus membrane**	Visual	Observe appearance of tissue	Pink and moist	Reddened or coated (increased whiteness) without ulcerations	Ulcerations with or without bleeding
**Gingiva**	Tongue blade and visual	Gently press tissue with tip of blade	Pink and stippled	Edematous with or without redness	Spontaneous bleeding or bleeding with pressure
**Teeth**	Visual	Observe appearance of teeth	Clean and no debris	Plaque or debris in localized area (between teeth if present)	Plaque or debris generalized along gum line

**Table 5:** Studies on cryotherapy, photobiomodulation (PBM), and usage of palifermin or caphosol.

Author	Number of Patients	Type of Study	Management and Treatment of Oral Mucositis
Askarifaret al. (2016) [43]	29 patients	Randomized study	Cryotherapy
Lu et al. (2020) [44]	145 patients	Prospective randomized study	Cryotherapy
Kamsvåg T et al. (2020) [45]	49 patients	Randomized study	Cryotherapy
Gantam et al. (2015) [52]	46 patients	Randomized study	PBM
Oton-Leite et al. (2015) [53]	30 patients	Randomized study	PBM
Antunes et al. (2013) [54]	94 patients	Randomized study	PBM
Henke et al. (2011) [61]	186 patients	Randomized study	Palifermin
Le et al. (2011) [62]	94 patients	Randomized study	Palifermin
Raphael et al. (2014) [66]	29 patients	Randomized study	Caphosol
